# Epigenetic silencing of CD4 expression in nonpathogenic SIV infection in African green monkeys

**DOI:** 10.1172/jci.insight.139043

**Published:** 2020-09-17

**Authors:** Joseph C. Mudd, Stephen Lai, Sanjana Shah, Andrew Rahmberg, Jacob K. Flynn, Carly E. Starke, Molly R. Perkins, Amy Ransier, Sam Darko, Daniel C. Douek, Vanessa M. Hirsch, Mark Cameron, Jason M. Brenchley

**Affiliations:** 1 Barrier Immunity Section, Laboratory of Viral Diseases, Division of Intramural Research,; 2 Human Immunology Section, Vaccine Research Center, and; 3 Nonhuman Primate Virology Section, Laboratory of Molecular Microbiology, Division of Intramural Research, National Institute of Allergy and Infectious Diseases, NIH, Bethesda, Maryland, USA.; 4 Department of Population and Quantitative Health Sciences, Case Western Reserve University, Cleveland, Ohio, USA.

**Keywords:** AIDS/HIV, Immunology, T cells

## Abstract

African green monkeys (AGMs) are natural hosts of SIV that postthymically downregulate CD4 to maintain a large population of CD4^–^CD8aa^+^ virus-resistant cells with Th functionality, which can result in AGMs becoming apparently cured of SIV_agm_ infection. To understand the mechanisms of this process, we performed genome-wide transcriptional analysis on T cells induced to downregulate CD4 in vitro from AGMs and closely related patas monkeys and T cells that maintain CD4 expression from rhesus macaques. In T cells that downregulated CD4, pathway analysis revealed an atypical regulation of the DNA methylation machinery, which was reversible when pharmacologically targeted with 5-aza-2 deoxycytidine. This signature was driven largely by the dioxygenase TET3, which became downregulated with loss of CD4 expression. CpG motifs within the AGM CD4 promoter region became methylated during CD4 downregulation in vitro and were stably imprinted in AGM CD4^–^CD8aa^+^ T cells sorted directly ex vivo. These results suggest that AGMs use epigenetic mechanisms to durably silence the CD4 gene. Manipulation of these mechanisms could provide avenues for modulating SIV and HIV-1 entry receptor expression in hosts that become progressively infected with SIV, which could lead to novel therapeutic interventions aimed to reduce HIV viremia in vivo.

## Abstract 2

African green monkeys have co-evolved with SIVagm via differential methylation of regulatory regions of the CD4 gene resulting in T cells that are resistant to SIV infection in vivo.

## Introduction

African green monkeys (AGMs) (genus *Chlorocebus*) are a natural host of SIV (SIV_agm_). AGMs maintain high viral loads throughout the disease course yet do not progress to simian AIDS. Lack of pathogenicity is not determined by virus-intrinsic factors, as SIV_agm_ is pathogenic in experimentally infected pigtail macaques (*Macaca nemestrina*) (1, 2). Host factors are thought to be important for the nonprogressive nature of SIV_agm_ infection in AGMs. These host factors include (but are not limited to) maintenance of the immune and structural components of the gastrointestinal tract (3, 4), robust yet transient induction of innate immune responses to SIV (5, 6), and lower viral burden in lymphoid tissues (7). Each of these characteristics is thought to contribute to a lack of chronic immune activation in natural hosts, an accurate predictor of disease progression in untreated HIV-1–infected humans and SIV-infected Asian macaques (8, 9).

An additional feature of natural hosts is their remarkable ability to regulate expression of the HIV-1/SIV host entry receptors CD4 and CCR5 (10, 11). Healthy adult AGMs, in particular, maintain low CD4 T cell counts (0–400 cells/μl) and a large pool of CD4^–^CD8αα^+^ memory T cells (10, 12, 13). CD4^–^CD8αα^+^ T cells in AGMs originate from canonical CD4^+^ T cells that have postthymically downregulated CD4 in vivo (10, 13). Thus, the functional profile of these cells exhibits clear distinctions from that of classical CD8αβ T cells and also hallmark similarities to the Th lineage, including MHC class II restriction; expression of FoxP3, CD40 ligand; and the ability to produce IL-17 and/or IL-2 (10, 13, 14). Importantly, CD4^–^CD8αα^+^ T cells are refractory to SIV infection in vivo (10), a phenotype that is closely shared by virus-resistant Th-like populations described in other natural hosts (15–17).

While very little is known regarding the mechanisms of CD4 postthymic repression in AGMs, the process can be readily studied in vitro, as CD4 expression in AGMs is exceptionally unstable. Multiple stimuli can induce AGM CD4 T cells to downregulate CD4, including antigen or homeostatic cytokines (13, 18). It is thought that events governing CD4 downregulation are inherently linked to those involved in cell division. Moreover, CD4 mRNA levels in AGM CD4^+^ T cells decline dramatically upon stimulation in vitro and are virtually absent in CD4^–^CD8αα^+^ T cells (10, 19), implying that loss of CD4 at the cell surface is the result of transcriptional repression and not mediated posttranslationally.

There are rare instances when AGMs will drive this mechanism to completion. In a previous study, one AGM with detectable blood CD4 counts upon importation subsequently converted its entire CD4^+^ T cell pool to a CD4^–^CD8αα^+^ phenotype (10, 13). Consequently, this animal became aviremic and evidently cured itself of SIV infection (10, 13). A similar case has been reported in a patas monkey, which exhibited no signs of immunodeficiency and yet had undetectable CD4 counts and was resistant to SIV_agm_ exposure (17). These instances highlight the critical link between SIV host entry receptor expression and viremia. Thus, understanding mechanisms of CD4 postthymic repression in natural hosts could have translational value in settings of progressive HIV-1/SIV infections. In this study, we used a whole-genome transcript-wide approach to understand the key molecular events driving CD4 downregulation in AGM T cells.

## Results

### Genes uniquely regulated in natural host CD4 T cells induced to downregulate CD4 in vitro.

**Figure 1:**
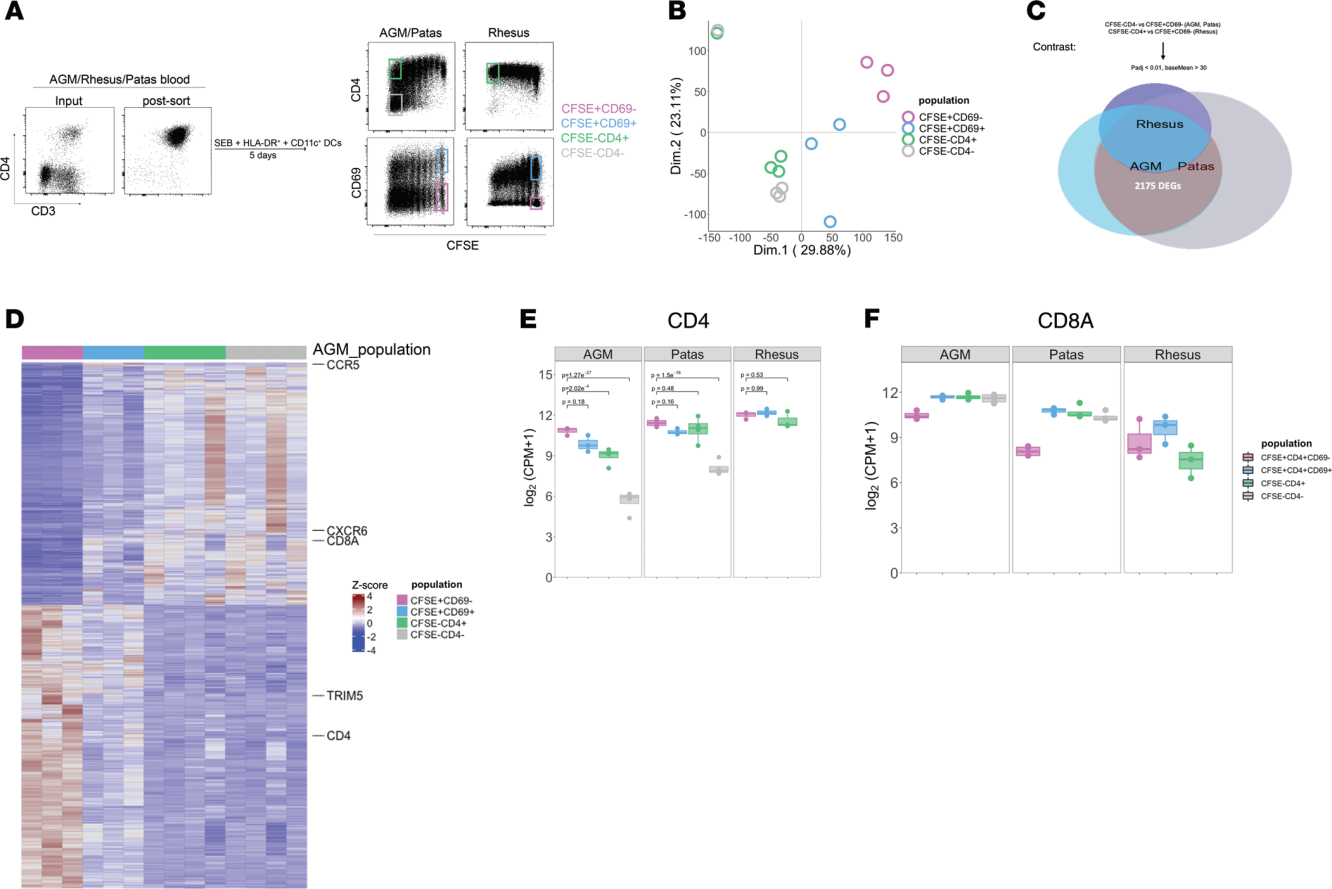
Uniquely regulated genes in natural host CD4^+^ T cells induced to downregulate CD4 in vitro. (**A**) Representative flow dot plot of purified AGM, patas, or rhesus macaque CD4^+^ T cells that were exposed to staphylococcus enterotoxin B and HLA-DR^+^CD11c^+^ antigen-presenting cells for 5 days. (**B**) Principal component analysis plot of transcribed genes from AGM CFSE^+^CD69^–^ (*n* = 3), CFSE^+^CD69^+^ (*n* = 3), CFSE^–^CD4^+^ (*n* = 4), CFSE^–^CD4^–^ (*n* = 4) T cell populations, based on transcript counts per million (CPM), calculated by Deseq2. The numbers in parenthesis on each axis represent the percentage of variance that each principle component contributes to the data set. (**C**) Euler diagram of DEGs in pairwise comparisons of CFSE^–^CD4^–^ (AGM, patas) or CFSE^–^CD4^+^ (Rhesus) cells versus the CFSE^+^CD69^–^CD4^+^ population of each species. Significant DEGs were calculated by the Wald test and corrected for multiple comparisons (Benjamini-Hochberg) in Deseq2. (**D**) Heatmap depicting transcript counts of genes common to natural hosts yet unique from rhesus, normalized by row *Z*-score. Genes known to play a role in disease nonprogression are annotated. Red and blue coloring represent genes that are upregulated and downregulated, respectively. Statistical comparisons of (**E**) CD4 and (**F**) CD8A log among sorted populations of AGM, patas, and rhesus. Statistical significance was calculated by the Wald test and corrected for multiple comparisons (Benjamini-Hochberg) in Deseq2**.**

To define genes linked to CD4 repression in natural hosts, we took advantage of the fact that African green and patas monkey T cells downregulate CD4 when stimulated in vitro, whereas rhesus macaque T cells do not. CFSE-labeled peripheral blood CD4^+^ T cells of each nonhuman primate species were flow cytometrically sorted and cocultured with autologous CD11b^+^HLA-DR^+^ dendritic cells in the presence of the super antigen staphylococcal enterotoxin B (SEB). As expected, SEB stimulation induced CD4^+^ T cells from African green (*Chlorocebus pygerythrus* i.e., vervet) and patas monkeys (*Erythrocebus patas*) to downregulate CD4 with subsequent divisions, yet CD4 expression on rhesus macaque (*Macaca mulatta*) CD4^+^ T cells remained stable (Figure 1A). We reasoned that genes linked to CD4 downregulation in natural host species could be tracked as CD4 T cells respond to stimulation. Thus, after 5 days in culture, we performed genome-wide transcriptome sequencing on cell populations of 4 distinct phenotypes: resting undivided (CD69^–^CFSE^+^) and activated undivided (CD69^+^CFSE^+^) (both of which express CD4), divided cells that retained CD4 (CFSE^–^CD4^+^), and divided cells that lost CD4 (CFSE^–^CD4^–^) (Figure 1A). Principal component analysis (PCA) revealed that gene expression profiles of CFSE^+^CD69^–^, CFSE^+^CD69^+^, and CFSE^–^ populations in AGMs clustered distinctly along the PC1 axis (Figure 1B). In contrast, gene expression profiles of CFSE^–^CD4^+^ and CFSE^–^CD4^–^ populations differed only slightly along the more minor second principle component (Figure 1B), suggesting that CD4 downregulation is likely the result of pathways comprising a distinct subset of genes.

We next performed pairwise comparisons of natural host CFSE^–^CD4^–^ or rhesus CFSE^–^CD4^+^ gene expression profiles with that of resting CFSE^+^CD69^–^ cells. The additional parameter of CFSE was included in this comparison because division is inherently coupled to the process of CD4 downregulation, and signaling pathway alterations controlling CD4 may precede loss of CD4 protein at the cell surface. This analysis revealed sets of significant differentially expressed genes (DEGs) that were both common and unique among each particular species. Of these DEGs, 2175 were found to be commonly expressed in CFSE^–^CD4^–^ cells of AGMs and patas monkeys and yet distinct from those of rhesus CFSE^–^CD4^+^ cells (Figure 1C). We reasoned that genes reported to play putative roles in disease nonprogression of natural hosts would be present in this set of genes. Indeed, CXCR6, the preferred coreceptor of SIV_agm_ that is thought to divert replication from more stem-like CD4^+^CCR5^+^ central memory cells (20, 21), was uniquely upregulated in CFSE^–^CD4^–^ cells of AGMs and patas monkeys and yet not in rhesus CFSE^–^CD4^+^ cells (Figure 1D). Importantly, CD4 was one of the most significant DEGs that was unique to CFSE^–^CD4^+^ cells (Figure 1D), as African green and patas (but not rhesus) CD4^+^ T cells exhibited significant loss of *CD4* transcription in CFSE^–^CD4^–^ cells (Figure 1E). *CD8A* transcript levels in the 2 natural host species significantly increased beginning at the CFSE^+^CD69^+^ stage (Figure 1F), suggesting that the pathways regulating the *CD4* and *CD8A* genes are distinct and occur at different kinetics.

### DNA methylation proteins contribute to CD4 gene silencing in natural hosts.

**Figure 2:**
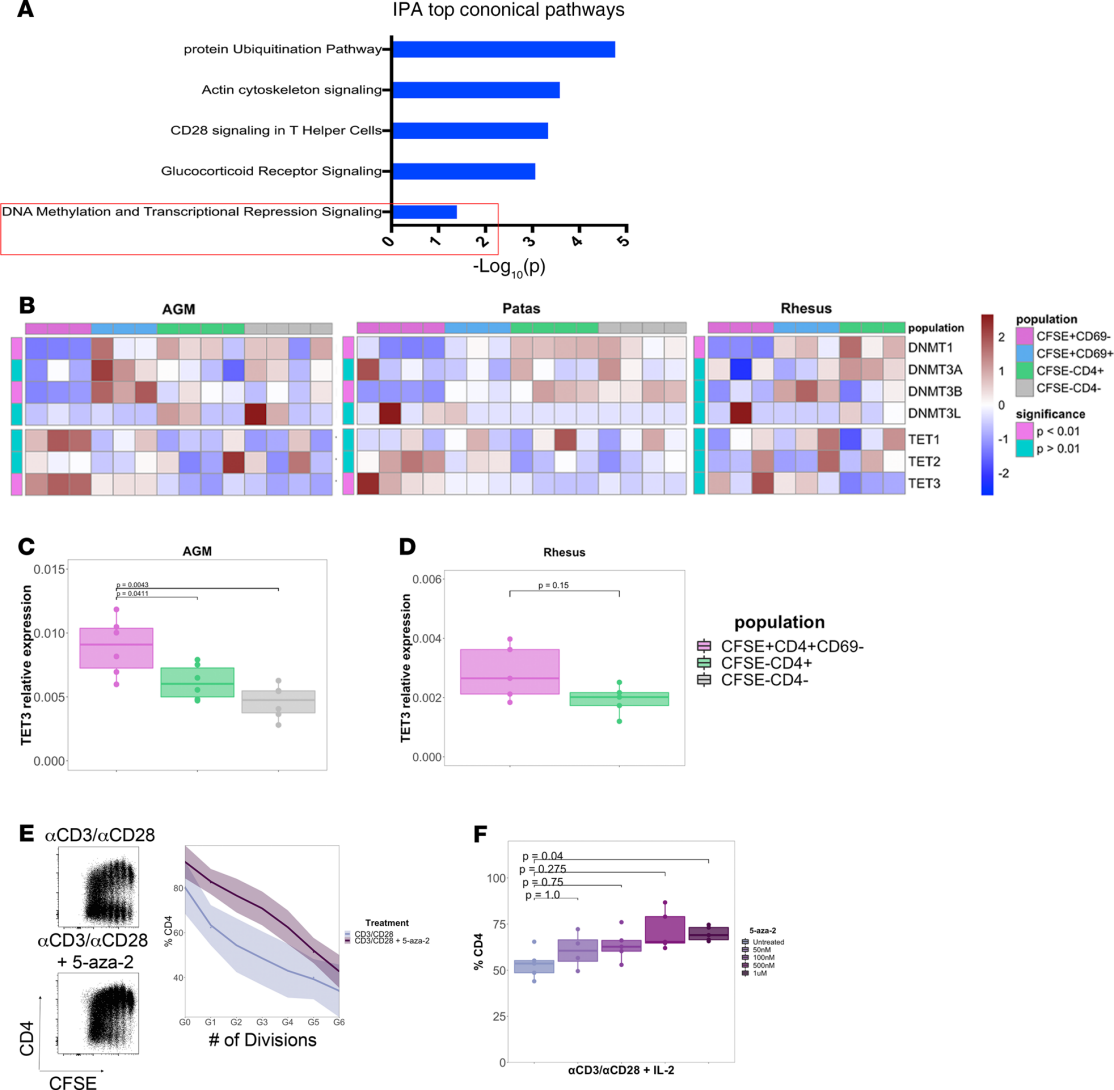
DNA methylation pathways contribute to CD4 gene silencing in natural hosts. (**A**) Top 5 canonical pathways from Ingenuity Pathway Analysis (IPA) of genes unique to natural host cells that are induced to downregulate CD4. *P* values were calculated by Fisher’s exact test. (**B**) Heatmaps depicting transcript abundance of genes encoding for proteins involved in DNA methylation of the 3 nonhuman primate species. The scale represents transcript counts normalized by row *Z*-score. Red and blue coloring represents upregulated and downregulated gene expression, respectively. Comparisons of TET3 gene expression in (**C**) AGM (*n* = 6) and (**D**) rhesus (*n* = 5) relative to GAPDH assessed by real-time PCR. Statistical significance was calculated by the Mann-Whitney test. (**E**) Representative flow dot plot and summary data (*n*= 6) depicting CD4 expression on AGM CD4^+^ T cells stimulated with anti-CD3/CD2/CD28 microbeads and 25 U/mL IL-2 for 5 days in the presence or absence of 500 nM 5-aza-2 deoxycytidine. (**F**) Dose response of 5-aza-2 deoxycytidine on CD4 downregulation in AGMs (*n*= 5). Significance in was determined by the Mann-Whitney test with multiple comparison adjustment by Bonferroni correction.

Given that CD4 transcripts were uniquely downregulated in natural host CFSE^–^CD4^–^ cells, we reasoned that gene networks upstream of CD4 silencing would also be uniquely regulated in this population. Thus, we analyzed the 2175 genes unique to natural host CFSE^–^CD4^–^ cells against a priori defined gene networks in the Ingenuity Pathway Analysis (IPA) database to determine pathways that may be relevant to CD4 expression. Canonical pathways enriched in CFSE^–^CD4^–^ cells included genes encoding for proteins involved in protein ubiquitination, T cell costimulation, actin cytoskeletal signaling, and DNA methylation (Figure 2A). We reasoned that DNA methylation may be particularly relevant for CD4 silencing in AGMs, given its general role as an epigenetic mediator of gene repression and previous studies linking the DNA methylation machinery to CD4 gene silencing in murine cytotoxic CD8^+^ T cells (22, 23). Methylation of DNA occurring at the fifth carbon atom of cytosine residues (5mC) is mediated by the DNA methyltransferase (DNMT) proteins *DNMT1*, *DNMT3A*, and *DNMT3B*, which influence inherited and de novo methylation patterns, respectively (23). This process can be reversed by the action of ten eleven translocation (TET) methylcytosine dioxygenases (TET1, -2, -3), which progressively oxidize methylated CpG motifs to 5-hydroxymethylcytosine, 5-formylcytosine, and 5-carboxylcytosine (23). We thus examined dynamics of the DNA methylation machinery in the 4 distinct cellular states induced by SEB stimulation of AGM, patas, and rhesus monkey CD4^+^ T cells. Gene expression of *DNMT1* increased in response to SEB stimulation of CD4^+^ T cells in all 3 nonhuman primate species (Figure 2B). In contrast, only CD4^+^ T cells from natural host AGM and patas animals exhibited upregulation and downregulation of *DNMT3B* and *TET3* gene expression in response to SEB, respectively (Figure 2B). We confirmed by qPCR that *TET3* transcripts were uniquely downregulated in divided AGM cells that lose CD4 (Figure 2C) and yet not significantly so in divided rhesus CD4^+^ T cells (Figure 2D).

Given that DNMT-mediated 5mC deposition near regulatory regions of DNA promotes gene silencing, whereas the 5hmC products of TET activity are associated with actively transcribed regions, we reasoned that the balance of DNMT and TET activities could potentially influence transcription of CD4. Perturbing this balance may favor transcription versus repression. Thus, we sought to manipulate CD4 expression pharmacologically in AGMs with the DNMT inhibitor 5′-aza-2′-deoxycytidine (5-aza-2). AGM CD4^+^ T cells activated in vitro with anti-CD3/CD2/CD28 microbeads and recombinant IL-2 in the presence of 5-aza-2 downregulated CD4 to a significantly (*P* = 0.04) lesser extent than those receiving anti-CD3/CD28 alone, an effect that was observed across multiple generations of divided CD4^+^ T cells (Figure 2E) and was dose dependent (Figure 2F). These data demonstrate that components of the DNA methylation machinery are uniquely regulated and can be pharmacologically targeted in vitro to manipulate CD4 expression in natural hosts of SIV.

### CD4 downregulation is associated with methylation of the CD4 promoter in AGMs.

**Figure 3:**
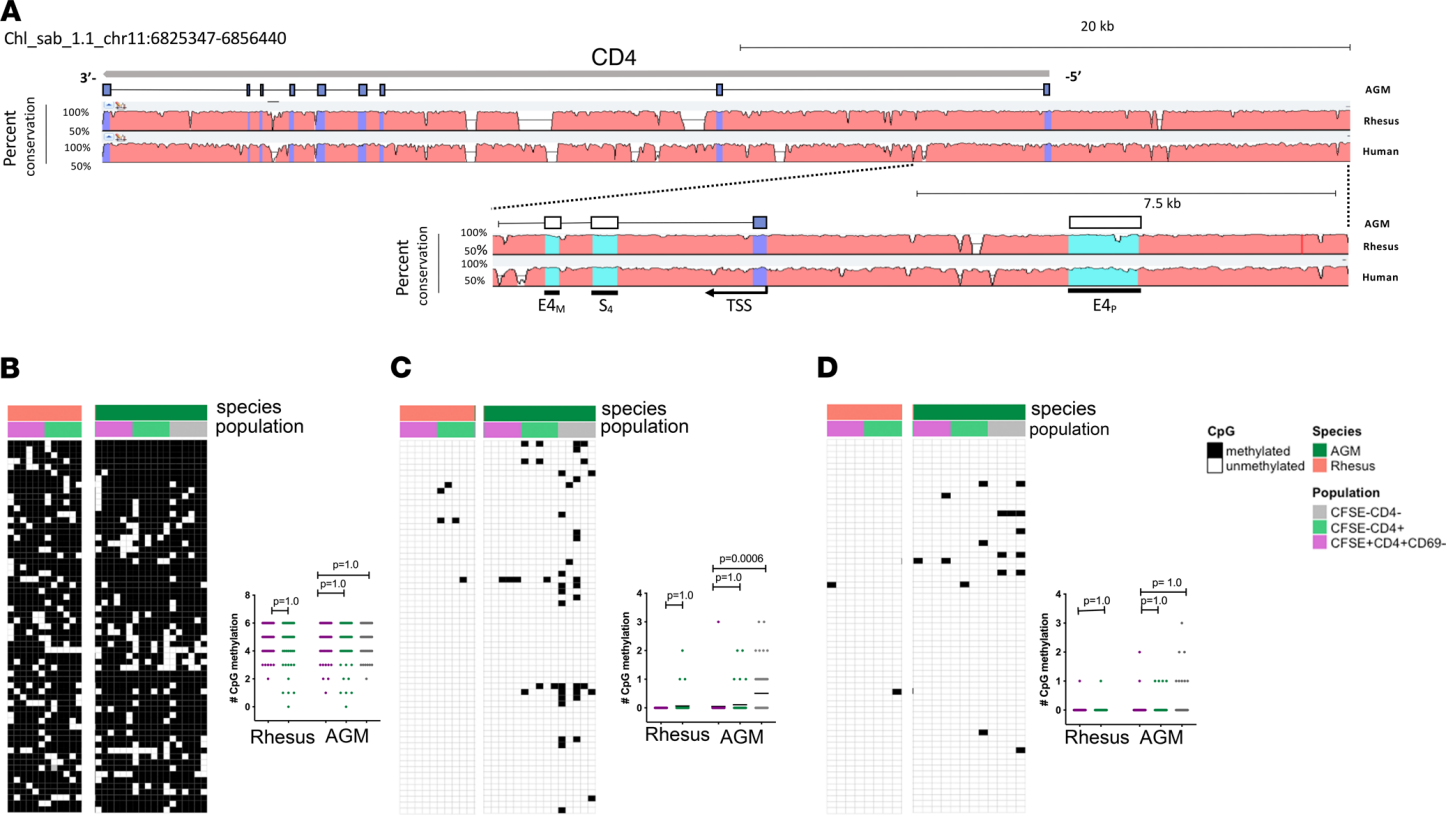
CD4 downregulation is associated with methylation of the CD4 promoter in AGMs. (**A**) Schematic representation of the *CD4* locus. Top: Entire CD4 gene locus of AGM + 10,000 base pairs upstream of the transcription start site, with percentage sequence conservation to rhesus and human. Bottom: 15,000–base pair region flanking the CD4 transcription start site encompassing the proximal enhancer (E4_p_), first exon, silencer (S_4_), and maturation enhancer (E4_M_). Percentage sequence conservation to rhesus and human is shown. Heatmaps and summary data of CpG methylation in regions amplified within the (**B**) E4_p_, (**C**) transcription start site, and (**D**) E4_p_ regulatory elements of CFSE^+^CD4^+^CD69^–^, CFSE^–^CD4^+^, and CFSE^–^CD4^–^ populations. Each column represents a single CpG dinucleotide found within the given region. Each row represents DNA from a single cell, totaling 63 individual clones from 3 individual animals. Significance in summary data was determined by the Mann-Whitney test with multiple comparison adjustment by Bonferroni correction.

Multiple *cis*-acting genomic elements proximal to or within the CD4 gene locus regulate CD4 expression in developing thymocytes. These include an upstream 430-nucleotide sequence known as the CD4 proximal enhancer (E4_p_) (essential for promoting CD4 expression in CD4^–^CD8^–^ double-negative [DN] thymocytes; refs. 24, 25), an intronic CD4 silencer (S_4_) (important for repressing CD4 in DN T cells and for initiating the CD4-silenced state of mature cytotoxic CD8^+^ T cells; refs. 26, 27), and a second intronic “maturation” enhancer (E4_M_) (in concert with E4_p_, regulates CD4 expression in late-stage CD4^+^, single-positive thymocytes) (28, 29). We sought to probe the genomic region encoding for CD4 in AGMs, finding that the structure of the CD4 locus, including genic and regulatory regions in AGMs, is highly homologous to humans and rhesus (Figure 3A). We further performed genomic alignments of the CD4 regulatory elements among AGMs, patas monkeys, rhesus macaques, and humans, postulating that shared sequences of AGMs and patas monkeys diverging from conserved sequences of rhesus and humans may have the potential to be significant. All CD4 regulatory regions were generally well conserved in each species, with sequence homology of 98% in all species (Supplemental Figure 1; supplemental material available online with this article; https://doi.org/10.1172/jci.insight.139043DS1). Nevertheless, a small number of nucleotide variations unique to natural hosts were present in the E4_p_, S_4_, and E4_M_ regulatory regions (Supplemental Figure 1), suggesting a degree of variation in *cis*-regulating factors of the CD4 locus of natural hosts.

Given that unique downregulation of *TET3* in AGMs could potentially influence CpG methylation state within the CD4 locus, we next performed bisulfite sequencing to assess differentially methylated regions (DMRs) within approximately 150–base pair regions of positive CD4 genomic regulatory elements (E4_P_, transcription start site [TSS], E4_M_) in blood AGM and rhesus CD4^+^ T cells stimulated in vitro with CD3/CD28/CD2 microbeads and IL-2. The majority of CpG motifs within the assessed E4_P_ region of both species were methylated in CFSE^+^CD4^+^CD69^–^ cells and remained so during division (CFSE^–^CD4^+^) or CD4 downregulation (CFSE^–^CD4^–^) (Figure 3B). Within the CD4 TSS, CpG motifs were rarely methylated in CFSE^+^CD4^+^CD69^–^ and CFSE^–^CD4^+^ T cells of both species, and yet they became differentially methylated in AGM cells that were induced to downregulate CD4 (Figure 3C). Similar trends were observed downstream at the intronic E4_M_ regulatory element: sparse CpG methylation in CFSE^+^CD4^+^CD69^–^ and CFSE^–^CD4^+^ T cells and differential methylation in AGM cells that downregulated CD4 in vitro (Figure 3D). These results suggest that DMRs within the CD4 locus are site specific and increase in frequency at transcribed regions only when AGM cells lose CD4 expression, yet not in states where CD4 expression is maintained.

### CpG methylation patterns are stably inherited in AGM CD4^–^CD8αα^+^ T cells.

**Figure 4:**
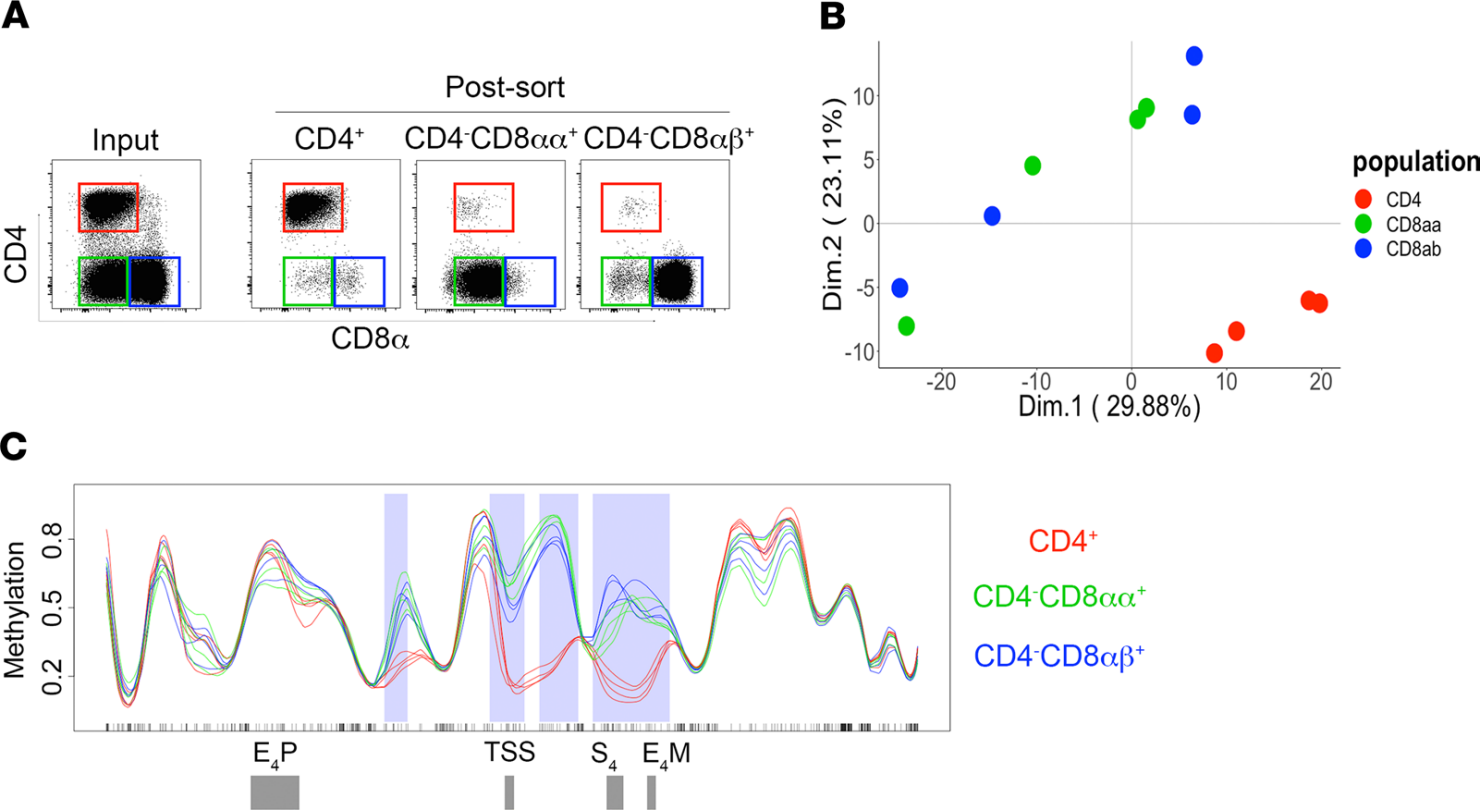
CpG methylation patterns are stably inherited in AGM CD4^–^CD8αα^+^ T cells. (**A**) Representative before and after sort flow dot plots of major T cell populations from AGM splenic samples. (**B**) Principal component analysis plot of AGM CD4 (*n* = 4), CD4^–^CD8αα^+^ (*n* = 4), CD4^–^CD8αβ^+^ (*n* = 4) methylation profiles of CD4 locus capture region, based on CpG methylation frequency determined by Bismark bisulfite read mapper. The numbers in parentheses on each axis represent the percentage of variance that each principle component contributes to the data set. (**C**) CpG methylation frequency across CD4 locus capture region, depicted by genomic coordinate. Coordinates of CD4 regulatory regions are annotated on bottom track. Shaded areas represent regions of hypermethylation when compared with methylated CpG frequencies in AGM CD4 T cells. Significance was determined by Fisher’s exact test with values listed in Supplemental Table 1.

The establishment of DMRs at the CD4 locus during in vitro CD4 downregulation led us to hypothesize that similar methylation patterns are stably inherited in AGM CD4^–^CD8αα^+^ T cells, which arise from mature CD4^+^ T cells that have durably silenced CD4 in vivo (10). Thus, we sorted CD4^+^, CD4^–^CD8αα^+^, and classical CD8αβ^+^ T cells to high purity from spleens of AGMs and assessed CpG methylation in regions proximal or within the CD4 locus (Figure 4A). To assess DMRs in more detail, we performed high-throughput sequencing of a capture region spanning 10,000 base pairs upstream and 10,000 base pairs downstream of the CD4 TSS, which encompassed all regulatory DNA elements (E4_P_, TSS, S_4_, E4_M_), the first exon, and majority of the first CD4 intron. PCA of CpG methylation frequency within the capture region revealed clear distinctions among the sorted T cell populations. Despite their functional similarity, CD4^–^CD8^+^ T cells clustered distinctly from CD4^+^ T cells and instead bared a striking resemblance to methylation profiles of classical CD8αβ^+^ T cells (Figure 4B). When CpG methylation frequencies of sorted populations were compared across genomic coordinates, methylation profiles of CD4^–^CD8αα^+^ T cells were virtually identical to those of classical CD8αβ^+^ T cells; however, they diverged considerably from those of CD4^+^ T cells, particularly within the CD4 gene body. Regions of marked hypermethylation in CD4^–^CD8^+^ T cells included 1 DMR approximately 3000 base pairs upstream of the CD4 TSS and 3 DMRs beginning at the CD4 TSS and extending approximately 5000 base pairs into the CD4 transcribed region, encompassing the first exon, S_4_ and E4_M_ regulatory elements (Figure 4C and Supplemental Table 1). Taken together, these data reveal that hypermethylation at distinct genomic locations within the AGM CD4 locus is associated with durable CD4 gene silencing in CD4^–^CD8αα^+^ T cells.

## Discussion

Repression of HIV-1/SIV entry receptor expression is a well-established feature of natural hosts (10, 11, 15, 30). In particular, negative regulation of host CD4 expression is both durable and irreversible, evidenced by anecdotal reports of healthy AGMs that convert their entire CD4^+^ T cell pool to a CD4^–^CD8αα^+^ phenotype and maintain a CD4 count of 0 throughout life (13, 14, 17). We took an unbiased approach to identify mechanisms of CD4 downregulation in AGMs, a process that allows canonical CD4 T cells to become resistant to SIV infection in vivo. An atypical regulation of genes encoding for proteins involved in DNA methylation characterized CD4 mRNA downregulation in T cells of 2 natural host species, which was not observed in rhesus macaques, with CD4 expression that remained stable. This signature was driven largely by unique reductions in expression of the dioxegenase *TET3* gene. We concurrently observed the CD4 promoter region in AGMs to become hypermethylated during CD4 downregulation in vitro. These observations remained highly consistent in spite of some heterogeneity in animal demographics or SIV serostatus. The CD4 promoter was also found to be hypermethylated in AGM CD4^–^CD8αα^+^ T cells, with durable in vivo CD4 repression and in contrast to AGM cells induced to lose CD4 in vitro, increased methylation extended well into the CD4 gene body. The stark difference in intragenic methylation between AGM cells that recently downregulated CD4 in vitro versus AGM CD4^–^CD8αα^+^ T cells is suggestive of promoter methylation occurring early in the process of CD4 downregulation, with gene body methylation occurring at later time points. These data suggest an epigenetic basis for HIV-1/SIV receptor control in natural hosts.

Transcriptional control of the CD4 locus has been most extensively studied during T cell development, a period at which thymocytes must actively regulate CD4 expression to ensure error-free lineage decisions. The current model suggests that *cis*-acting enhancer elements proximal CD4 enhancer (E4_p_) and maturation CD4 enhancer (E4_M_) cooperate with *trans*-acting factors to drive optimal CD4 expression during positive selection and to stabilize expression in proliferating mature CD4^+^ T cells (29, 31). Interestingly, there is a clear temporal basis for the activity of these elements. In mice, germline deletion of E4_p_ or E4_M_ results in significantly lower CD4 thymic output and unstable expression in proliferating mature CD4^+^ T cells (28, 29). In contrast, when these elements are deleted in mature CD4 T cells (after thymic selection) expression of CD4 during proliferation is stably maintained (28, 32). There is thus a striking parallel to proliferating CD4^+^ T cells of AGMs and those of germline E4_M_^–/–^ or E4_p_^–/–^ mice, in that CD4 expression in both instances is unstable, raising the possibility that loss-of-function mutations to regulatory regions active during T cell development may subsequently effect stability of CD4 in mature AGM T cells. We show here that AGMs and patas animals share a small number of nucleotide differences in the E4_M_ and E4_p_ enhancer elements that are unique from those of rhesus macaques or humans that exhibit stable CD4 expression. We cannot rule out that these mutations impact transcriptional regulation of CD4, although it is important to note that thymic CD4 output in African green and patas monkeys is normal, and juvenile AGMs exhibit CD4 T cell counts similar to those of rhesus macaques.

Our results are in accordance with DNA methylation playing an inhibitory role on CD4 transcription in AGMs. We cannot rule out the possibility that locus methylation is simply a consequence of reduced transcriptional activity by *trans*-acting factors that bind the CD4 promoter, presumably making the locus more permissive to methylation. However, the facts that CD4 locus methylation also occurs in proliferating CD4 T cells of E4_M_^–/–^ or E4_p_^–/–^ mice and that we can manipulate CD4 expression directly in AGMs with the DNMT inhibitor 5-aza-2 suggest that locus methylation plays a causative role in CD4 repression. Moreover, regions that become differentially methylated during CD4 downregulation in AGMs are highly site specific, occurring within the gene body but not in regions upstream of the CD4 TSS.

A contributing factor to locus hypermethylation may be the unique loss of *TET3* gene expression in proliferating AGM CD4 T cells. In other settings, loss of TET activity promotes locus hypermethylation and subsequently impacts the stability of gene expression. This is true of the *FOXP3* locus and, importantly, the CD4 locus (33, 34). Proliferating mature CD4^+^ T cells from TET1/TET3 double-knockout mice phenocopy the lack of CD4 stability observed in proliferating AGM CD4^+^ T cells (29).

Comparative studies in natural hosts have proven invaluable in dissecting the salient features of HIV-1/SIV pathogenesis, particularly at tissue sites (9). Our study sheds light on an additional use of natural hosts as potential guides for HIV eradication strategies. Indeed, control of SIV entry receptor expression in natural hosts has led to several documented instances of spontaneous SIV cure (13, 14, 17). Probing the mechanism of CD4 repression in AGMs raises the possibility of therapeutically altering these same pathways in progressive hosts. These include candidate targets that may influence CD4 expression (such as TET3) or editing approaches that manipulate CD4 directly. In a select number of HIV-1^+^ human subjects, analogous approaches have sought to purge the host of susceptible HIV targets by targeting CCR5. Two of these instances have been marked with ART-free long-term remission (35, 36). One incident however resulted in viral rebound of a CXCR4-tropic strain after ART discontinuation (37). Thus, approaches that seek to render cells virus resistant by alternatively targeting CD4 could presumably protect against X4-tropic breakthroughs and also widen therapeutic efficacy to subjects with more diverse viral reservoirs. This is notwithstanding significant caveats that would need to be addressed with this approach. CD4 serves to amplify TCR signaling by recruiting the protein tyrosine kinase Lck to the immunological synapse (38, 39), and blockade of CD4 significantly impairs T cell sensitivity to antigen (40, 41). Furthermore, expression of CD4 on monocytes and macrophages would remain unaffected in an approach that solely targets the lymphoid lineage. Although the precise role of macrophages and other myeloid cells as a bon a fide reservoir for HIV-1 during ART is still controversial (42–45), ablation of CD4 could conceivably result in the disregarding of nonlymphoid sources of HIV-1 that could potentially maintain the reservoir if ART is interrupted. Thus, questions remain on the feasibility of a strategy that could potentially be detrimental to classical Th functions. This dovetails with the related question raised by this study and others on the apparent dispensability of CD4 in AGMs. For example, AGMs can mount robust recall responses to MHC-II–restricted antigens in the CD4^–^CD8αα^+^ T cell compartment (13), and these animals do not suffer from any apparent immunodeficiency or autoimmunity. Whether AGMs have evolved mechanisms to compensate for the loss of CD4 remains to be determined. Nevertheless, these data highlight key mechanisms of HIV-1/SIV entry receptor control in natural hosts and potential avenues for their manipulation in settings of progressive infection.

## Methods

### Nonhuman primates.

This study was performed on 11 vervet AGMs, 4 patas monkeys, and 9 rhesus macaques. All relevant animal information including age, sex, and SIV status is summarized in Supplemental Table 2. If indicated, all experimental SIV infections were performed intravenously with SIVmac239 (rhesus) or SIV_agm_ (AGM).

All procedures were carried out under ketamine anesthesia by trained personnel under the supervision of veterinary staff, and all efforts were made to maximize animal welfare and to minimize animal suffering in accordance with the recommendations of the Weatherall report on the use of nonhuman primates in research (46). Animals were housed singly in adjoining individual primate cages, allowing social interactions, under controlled conditions of humidity, temperature, and light (12-hour-light/12-hour-dark cycles). Food and water were available ad libitum. Animals were monitored twice daily and fed commercial monkey chow, treats, and fruit twice daily by trained personnel. Environmental enrichment was provided in the form of primate puzzle feeders, mirrors, and other appropriate toys.

### In vitro cell culture for gene expression analysis.

For in vitro stimulation of samples used for RNA-Seq, fresh PBMC suspensions were isolated under Ficoll-Paque density gradient separation, labeled with 400 nM of CellTrace violet from a 400 μM stock concentration (Thermo Fisher Scientific), and sorted for CD4^+^ T cells on a BD FACSAria II. Cells were subsequently cultured in RPMI supplemented with 10% fetal bovine serum, 100 IU/mL Penicillin, 100 μg/mL Streptomycin, and 2.5 mM glutamine (all from Gibco) in the presence of 1 μg/mL SEB (Millipore Sigma) and autologous HLA-DR^+^CD11b^+^ antigen presenting cells at 10:1 ratios. After 5 days of culture, responding cells were resorted on the basis of CD4 expression, CFSE dye dilution, and CD69 expression; lysed in 350 μl RLT buffer supplemented with 1% β-2-mercaptoethanol (QIAGEN; and preserved at –80°C until further processing.

All experiments requiring in vitro stimulation shown in Figures 2 and 3 were performed with anti-CD3/CD28/CD2 microbeads. In brief, PBMCs from AGMs and rhesus nonhuman primate species were labeled with CellTrace Violet at the identical concentration as above (Thermo Fisher Scientific) and CD4 T cells were subsequently magnetically purified with anti-CD4 microbeads (Miltenyi Biotech). Cells were then stimulated in cRPMI with the nonhuman primate T cell activation/expansion kit (bead-immobilized antibodies to anti-CD3/CD2/CD28) (Miltenyi Biotech) at 1:2 bead-to-cell ratios in the presence of 25 U/mL IL-2 (R&D Systems) for 7 days. cRPMI media containing fresh IL-2 was replenished to cultures at days 2, 4, and 6. For experiments with the DNMT inhibitor 5-aza-2 deoxycytidine, CD4 T cells from AGMs were isolated and stimulated as above. At day 2 after stimulation, cells were treated with 500 nM 5-aza-2 deoxycytidine dissolved in DMSO (MilliporeSigma), and CD4 surface expression was measured at day 7.

### Cell phenotyping.

T cell phenotyping for all experiments was assessed with antibodies reactive to the following surface antigens: anti-CD3 Alexa Fluor 700 (clone SP43-2, catalog 557917), anti-CD4 Allophycocyanin (clone L200, catalog 551980), anti-CD8α Pacific Blue (clone RPA-T8, catalog 558207), and anti-CD69 Phycoerythrin.cy7 (PE-cy7) (clone FN50, catalog 557745) (all from BD). Cells were washed once in ice-cold PBS and incubated with fluorochrome-labeled antibodies at 4°C with the addition of LIVE/DEAD amine-reactive viability dye (Thermo Fisher Scientific). After 20 minutes, cells were washed a second time in PBS. For experiments requiring only strict phenotyping, cells were fixed with 1% PFA and acquired on a BD FACS Fortessa.

### Library preparation and RNA-Seq.

For samples used for RNA-Seq in Figure 1, total RNA was isolated following the QIAGEN RNeasy Micro Kit protocol. Samples were purified through QIAGEN columns with on-column DNase treatment for 15 minutes. Libraries were made from purified total RNA using the Illumina TruSeq Stranded Total RNA Sample Preparation Kit protocol. rRNA was removed from samples by denaturing and then purified on rRNA removal beads. RNA was then fragmented and primed with random hexamers for first-strand synthesis in a single step on a thermocycler for 8 minutes at 94°C. First-strand synthesis immediately followed use of Superscript II polymerase (Invitrogen) on a thermocycler with the following parameters: 25°C for 10 minutes, 42°C for 15 minutes, and 70°C for 15 minutes. In order to determine which strand the transcript was transcribed from, dUTP’s were used instead of dTTP’s during the second-strand synthesis. Single adenines were added to the blunted double-stranded cDNA. Illumina adapters containing unique dual-indices were ligated to each library. This allowed the samples to be multiplexed on the sequencer. Libraries containing Illumina adapters were enriched using 15–18 cycles of PCR. Throughout multiple steps of the library preparation, oligonucleotide integrity was assessed by capillary electrophoresis with an Agilent DNA bioanalyzer instrument and DNA high-sensitivity chips. Libraries were sequenced on an Illumina HiSeq 2500. Data are available at the Gene Expression Omnibus (GEO) (accession GSE151815).

### Alignment and differential gene expression.

Files containing sequence reads and corresponding quality scores from nonhuman primate samples (rhesus, AGM, patas) specified for RNA-Seq in Supplemental Table 2 were aligned to the *Mmul_8.0.1* rhesus macaque genome assembly and annotation with STAR (ver2.7.3a) at default parameters (47). Bam files were sorted by genomic coordinate with SamTools (48) and subsequently counted with HTseq (49). Pairwise comparisons of gene counts (including normalization) were performed with DEseq2 (50), and all genes with a logCPM <30 were omitted from any downstream analysis. A *P* value of less than 0.01 when corrected for multiple comparison testing (Benjamini-Hochberg) was used to define genes as significantly differentially expressed. Refseq numbers of AGMs, rhesus macaques, and human genomes used for alignments in this study are as follows (in respective order): GCF_000409795.2, GCF_003339765.1, and GCF_000001405.39.

### Pathway and tertiary analysis.

Pathway analysis on the data set containing genes unique to natural host cells in the CFSE^–^CD4^–^ population was performed by IPA software (QIAGEN). Expression of particular genes was visualized with heatmaps generated by the package in R, “Pheatmap.” PCA calculations were performed with the R package “FactomineR.”

### Gene validation by qRT-PCR.

Cells from sorted AGM and rhesus T cell populations stimulated for 7 days in vitro with αCD3/CD28/CD2 microbeads and 25 ng/mL recombinant IL-2 were lysed with 350 μl RLT buffer, and RNA was subsequently isolated by silica membrane adsorption with the QIAGEN Allprep RNA/DNA isolation kit. Total RNA was quantified by Qubit (Thermo Fisher Scientific), and reverse transcription was performed with the high-capacity RNA-to-cDNA kit with equal amounts of RNA for each sample (Applied Biosystems). TET3 cDNA was amplified with species-specific intron-spanning primers noted in Supplemental Table 2 and PowerUp SYBR green master mix (Thermo Fisher Scientific), and reactions were carried out with the StepOne Plus Real-Time PCR machine (Applied Biosystems). GAPDH expression was used as a reference gene and amplified with primers specified in Supplemental Table 3.

### Bisulfite sequencing of single-cell clones.

To assess CpG methylation in single cells, genomic DNA from sorted populations indicated in Figure 3 was isolated with the QIAGEN Allprep RNA/DNA isolation kit and deaminated with the EZ DNA methylation lightning kit (Zymo Research). Bisulfite-converted DNA was eluted in water and regions specific to the E4_p_, TSS, and E4_M_ CD4 regulatory regions were amplified with species-specific primers complimentary to bisulfite-treated templates (Supplemental Table 3). PCR amplicons were subsequently ligated into pGEM Easy-T vectors (Promega) overnight at 4°C. Vectors were transformed into DH10B competent cells and, after overnight incubation at 37°C, individual colonies taking up the cloned inserts were selected onto agarose plates containing 100 μg ampicillin and IPTG/X-gal. A minimum of 30 clones was selected from each sorted cell population and amplified on a 96-well plate with exTaq enzyme (Takara) and primers specific for M13 sequences flanking the PCR cloning site (Supplemental Table 3). Individual clones were sequenced by Sanger sequencing (Eurofins Genomics).

### Custom-capture bisulfite sequencing.

To assess CpG methylation across the 20 kb region encompassing CD4 regulatory regions, genomic DNA from CD4, CD4^–^CD8αα, and CD8αβ T cells of cryopreserved AGM splenic cell suspensions was isolated using the QIAGEN allprep DNA/RNA isolation kit (QIAGEN). Splenic cell suspensions were previously prepared by mincing splenic tissue in 2 × 2 mm blocks and mechanically it digesting through a 50 μM mesh filter; this was followed by a 10-minute treatment with ammonium-chloride potassium treatment to lyse red blood cells and 2 subsequently washings in ice-cold 1× PBS. At least 3 μg genomic DNA was sheared with a Covaris sonicator to 100–175 nt fragment sizes. Library preparation was then followed in exact accordance to the Agilent methylseq library prep protocol. In brief, DNA libraries were prepared first by repairing and dA-tailing the 3′ end of the DNA fragments. Methylated adapters were subsequently ligated onto the DNA fragments. Genomic DNA libraries were then hybridized with proprietary hybridization probes designed by Agilent to be complimentary to the 20 kb capture region of the CD4 locus and incubated overnight at 65°C. Hybridized fragments were then captured with streptavidin beads, washed, and subsequently deaminated with the EZ DNA methylation lightning kit (Zymo Research). Bisulfite-converted libraries were indexed, amplified with 12 PCR cycles, and subsequently pooled for sequencing on an HTseq 4000 (Illumina). Following sample demultiplexing, reads were quality checked with command-line versions of FastQC and Cut-adapt and aligned to genomic coordinates of the capture region in the AGM genome (chr11:6815343-6835391, version ChlSab 1.1) with Bismark bisulfite read mapper (51). Coverage files containing CpG methylation frequency of the capture regions were further processed with the R package Bsseq (52).

### Statistics.

Statistical analysis for pairwise comparisons of RNA-Seq read counts were performed by the Wald test in the DEseq2 package in R. Two-group comparisons, including qPCR relative expression data and methylated CpG counts of single-cell clones, were performed in Prism version 6.0 (GraphPad) using the nonparametric Mann-Whitney test and subsequently corrected for multiple comparisons by the Bonferroni method. To assess DMRs in the custom-capture CD4 regulatory region, a Fisher’s exact test was performed for each individual CpG coordinate with the R package Bsseq. *P* values of less than 0.05 were considered significant.

### Study approval.

All animal work was approved by the National Institute of Allergy and Infectious Diseases Division of Intramural Research Animal Care and Use Committees (protocols LMM-6 and LVD-26). The animal facility is accredited by the American Association for Accreditation of Laboratory Animal Care. This study was carried out in strict accordance with the recommendations described in the *Guide for the Care and Use of Laboratory Animals* (National Academies Press, 2011).

## Author contributions

JCM and JMB designed the experiments. JCM, SL, SS, MRP, and A. Rahmberg performed experiments and analyzed data. JCM performed bioinformatic analysis. JKF and CES provided helpful protocol advice. A. Ransier, SD, DCD, and MC provided resources for whole-genome sequencing. VMH provided animal resources. All authors wrote the manuscript.

## Supplementary Material

Supplemental data

Supplemental Table 1

Supplemental Table 2

Supplemental Table 3

## Version 1 08/25/2020

In-Press Preview

## Version 2 09/17/2020

Electronic publication
