# Supplementary material for: Epigenetic silencing of CD4 expression in nonpathogenic SIV infection in African green monkeys
Source: JCI Insight. 2020 Sep 17;5(18):e139043. doi: 10.1172/jci.insight.139043 (PMC7526541; doi:10.1172/jci.insight.139043)
Supplement: Supplemental data [file jciinsight-5-139043-s176.pdf]

CLUSTAL O(1.2.4) multiple sequence alignment: E4P

```
Human       CACCTGTGGTCCTAGTTATTCAGGAGGCTGAGGGAGAAGAATCACTGGAGGCTAGGATTT    60
Rhesus      CACCTGTGGTCCTAGTTATTCAGGAGGCTGAGGGAGAGGAATCACTG----GAAGGATTT    56
Patas       CACCTGTGGTCCTAGTTATTCAGGAGGCTGAGGGAGAGGAATCACTGGAGCCCAGGATTT    60
AGM         CACCTGTGGTCCTAGTTATTCAGGAGGCTGAGGGAGAGGAATCACTGGAGCCCAGGATTT    60
            ************************************* *********      *******

Human       CCAGGCTGTAGTGAGCCATGATTGTGCTATTTCACTCCAGCTTGGGTGACAGGGTGAGAT    120
Rhesus      CCAGGCTGTAGTGAGCCATGATTGTGTTATTGCACTCCAGAGTGGGTGACAGGGTGAGAT    116
Patas       CCAGGCTGTAGTGAGCCATGATTGTGTTATTGCACTCCAGAGTGGGTGACAGGGTGAGAT    120
AGM         CCAGGCTGTAGTGAGCCATGATTGTGTTATTGCACTCCAGAGTGGGTGACAGGGTGAGAT    120
            ************************** **** ********  ******************

Human       CCTGTGTCTAAAAAGAATAATAACAATAATAATAAACATTATCTCACACAACGAGGAATC    180
Rhesus      CCTGTCTCTAAAAATAATAATAACGATCACAATACACACTATCTCACACAATGAGGAACT    176
Patas       CCTGTCTCTAAAAATAATAATAACGATCACAATACACACTATCTCACACAATGAGGAACT    180
AGM         CCTGTCTCTAAAAATAATAATAACGATCACAATACACACTATCTCACACAATGAGGAACT    180
            ***** ******** ********* ** * **** *** ************ ******
                                                          RUNX3
Human       CAGGAGCAGTCTTGCTGAGTAGTTCTGGCACAGGGTTGCTCATGAGGTTGCTGCTGAGGT    240
Rhesus      CAGGAGCAGTCTTGCTGAGTAGTTCTGGCACAGGGTCGCTCATGAGGTTGCTGCTGAGGT    236
Patas       CAGGAGCAGTCTTGCTGAGTAGTTCTGGCACAGGGTCGCTCATGAGGTTGCTGCTGAGGT    240
AGM         CAGGAGCAGTCTTGCTGAGTAGTTCTGGCACAGGGTCGCTCATGAGGTTGCTGCTGAGGT    240
            ************************************ ***********************

Human       GAGGGTCAGGTCTGGAGCCATCTGAAGGCTGGGATGGGGCTGGAGATTCTGCCTCCAAGA    300
Rhesus      GTGGGCCAGGCCAGGAGCCATCTGAAGGCTGGGATGGGGCTGGAGGATCTGCCTCCAAGA    296
Patas       GTGGGCCAGGCCAGGAGCCATCGGAAGGCTGGGATGGGGCTGGAGGATCTGCCTCCAAGA    300
AGM         GTGGGCCAGGCCAGGAGCCATTGGAAGGCTGGGATGGGGCTGGAGGATCTGCCTCCAAGA    300
            * *** **** * ********  **********************  *************

Human       CAGCTCACTCGGGAGGCTGTCGGCCAGAGGCCTTAACTCCTCCCTGT-GTGGGTGTCTTT    359
Rhesus      CAGCTCCCTTGGGAGGTTGTGGGCCAGAGGCCTTAGCGCCTCCCTGCGTTGGGCGTCTTT    356
Patas       CAGCTGCCCTGGGAGGTTGTGGGCCAGAGGCCTTAGCACCTCCCTGC-TTGGGCGTCTTT    359
AGM         CAGCTGCCCTGGGAAGTTGTGGGCCAGAGGCCTTAGCACCTCCCTGC-TTGGGCGTCTTT    359
            *****  *  **** * *** ************** * ********   **** ******

Human       ATGGGGCCTCTCACAACACGACACAGCAACTGGCTTCCCCCAGAGGGACAGCGTGGAAAA    419
Rhesus      ATGGGGCCTCTCACAACATGACACAGCAACTGGCTTCCCCCAGAGGGACAGCGTGGACAA    416
Patas       ATGGGGCCTCTCACAACACCACACGGCAACTGGCTTCCCTCAGAGGGACAGCGTGGACAA    419
AGM         ATGGGGCCTCTCACAACACCACACAGCAACTGGCTTCCCTCAGAGGGACAGCGTGGACAA    419
            ******************  **** ************** ***************** **
                                                 LEF1/TCF
Human       GACAAAAGCTACCACATCTTTATTTCAAGATTAGATTTGATCTTGAAAGCAACGTGCCAT    479
Rhesus      GACAAAAGCTACCATGTCTT----------------TTGATCTGGAAAACAACATACCAT    460
Patas       GACAAAAGCTACCATGTCTTTATTTCAAGATTGAATTTGATCTTGAAAACAACATACCAT    479
AGM         GACAAAAGCTACCATGTCTTTATTTCAAGATTGAATTTGATCTTGAAAACAACATACCAT    479
            **************  ****                ******* **** **** * ****

Human       AATTTCTGCCCTATTCTATTGATCACAGAGACCAACCTGGTGCAAGCAGGAGGGGGCAAT    539
Rhesus      GATTTCTGCCCTATTCTATTGGTCACAGAGACCAACCTGGTGCAAGCAGGAGGGGGCAAT    520
Patas       GATTTCTGCCCTATTCTATTGGTCACAGAGACCAACCTGGTGCAAGCAGGAGGGGGCAAT    539
AGM         GATTTCTGCCCTATTCTATTGGTCACAGAGACCAACCTGGTGCAAGCAGGAGGGGGCAAT    539
             ******************** **************************************

Human       GCAAGGGTGTGAACTGAGGGAGACAGGAGTCACTGGGGGCCATCTTGCAGCCTGGCTGTC    599
Rhesus      GCAAGGGTGTGAACGGAGGGAGACAGGAGTCACTGGGGGCCATCTTGCAGCCTGGCTGTC    580
Patas       GCAAGGGTGTGAACGGAGGGAGACAGGAGTCACTGGGGGCCATCTTGCAGCCTGGCTGTC    599
AGM         GCAAGGGTGTGAACGGAGGGAGACAGGAGTCACTGGGAGCCATCTTGCAGCCTGGCTGTC    599
            ************** ********************** **********************

Human       AGAGTGGGGAGCAGTGCCTGAAACAGAGATAGGCCAAGAGACGGGAAGGGACGTCTCTGA    659
Rhesus      AGAGTGGGGAGCAGTGCCTGAAACAGGGATAGGCCAAGAGAGGAGAAGGGACCCCTCTGA    640
Patas       AGAGTGGAGAGCAGTGCCTGAAACAGGGATAGGCCAAGAGAGGAGAAGGGACCCCTCCGA    659
AGM         AGAGTGGAGAGCAGTGCCTGAAACAGGGATAGGCCAAGAGAGGAGAAGGGACCCCTCCGA    659
            ******* ****************** ************** * ********  *** **

Human       GCATCTCCATCCCCAGCCCTCTTCTCTTGTCCACTCTCCAGAAGTTCTGGAAGCCAAGTG    719
Rhesus      GCATCTCCATCCCCAGCTGTCTTCTCTTGTCCACTCTCGAGAAGTTCTGGAAGCCAAGTG    700
Patas       GCATCTCCATCCCCAGCCATCTTCTCTTGTCCACTCTCGAGAAGTTCTGGAAGCCAAGTG    719
AGM         GCATCTCCATCCCCAGCCATCTTCTCTTGTCCACTCTCGAGAAGTTCTGGAAGCCAAGTG    719
            *****************  ******************* *********************

Human       AGGTCCAGGGCCTTGGCAAGTCGAGTGTGTGAGGTAGGAGGCCATACTGTGCACATTCAG    779
Rhesus      AGGTCTAGGGCCCTGGCAAGTCGAGTGTGTGAGGTAGAAGGCCGTACTGTGCACACTCAG    760
Patas       AGGTCTAGGGCCCTGGCAAGTCGAGTGTGTGAGGTAGGAGGCCGTACTGTGCACACTCAG    779
AGM         AGGTCTAGGGCCCTGGCAAGTCGAGTGTGTGAGGTAGGAGGCCGTACTGTGCACACTCAG    779
```

```
                ***** ****** ************************ ***** *********** ****

Human           CCTTGGAGTCGGGTTTGCCAGCGGTTCCCCTACCCCTGACGTGGTCTTCTCAGCTCCATA    839
Rhesus          CCCTGGAGTCGGGTTTGCCAGCTGTTCCCCTACCCGTGACCTGGTCTTCTCAGCTCCATA    820
Patas           CCTTGGAGTCGGGTTTGCCAGCCGTTCCCCTACCCGTGACCTGGTCTTCTCAGCTCCATA    839
AGM             CCCTGGAGTCGGGTTTGCCAGCCGTTCCCCTACCCGTGACCTGGTCTTCTCAGCTCCATA    839
                ** ******************* ************ **** *******************

Human           GTGCAGAGGAGGAGCCTCTGCAGGCCCAGAAATTTCCCTGCCCACCTCCCCTACCAGGTA    899
Rhesus          GCGCAGAGGAGGAGCCTCTGCAGGCCCAGAAATGTCCTTGCCCACCTCCCCTACCTGGTA    880
Patas           GCGCAGAGGAGGAGCCTCTGCAGGCCCAGAAATGTCCTTGCCCACCTCCCCTGCCTGGTA    899
AGM             GCGCAGAGGAGGAGCCTCTGCAGGCCCAGAAATGTCCTTGCCCACCTCCCCTGCCTGGTA    899
                * ******************************* *** ************** ** ****
                                                            LEF1/TCF
Human           GAGATGTTAGGGTTCAGGCCTGAGCCCCTGCTGCTATCCCTCCTTCAAAGGAGGAGATCA    959
Rhesus          GAGATGTTAGGGTTCAGGCCTGAGCCCCTGCTGCTATCCCTCCTTCAAAGGAGGAGATCA    940
Patas           GAGATGTTAGGGTTCAGGCCTGAGCCCCTGCTGCTATCCCTCCTTCAAAGGAGGAGATCA    959
AGM             GAGATGTTAGGGTTCAGGCCTGAGCCCCTGCTGCTATCCCTCCTTCAAAGGAGGAGATCA    959
                ************************************************************
                                                                 LEF1/TCF
Human           AGGAGCTTAGGGTCCCCCGCACAGGCCCACCCCAGGGTGGGGTTCTTCCTTTGAAGGGAA    1019
Rhesus          AGGAGCTTAGGATCCCCTGCACAGGCCCACCCCAGGGTGGGGTTCTTCCTTTGAAGGGAG    1000
Patas           AGGAGCTTAGGATCCCCTGCACAGGCCCACCCCAGGGTCGGGTTCTTCCTTTGAAGGGAA    1019
AGM             AGGAGCTTAGGATCCCCTGCACAGGCCCACCCCAGGGTGGGGTTCTTCCTTTGAAGGGAA    1019
                *********** ***** ******************** ********************
                                                   RUNX1
Human           TTGCTTTGGGGTGGGGTCGGTTCTATCTGCTCCACTCTGTGGCTGACAGTTTCTCCAAGG    1079
Rhesus          TGGGTTTGGGGTAGGGTCGGTTCTATCTGTTCTACTCTGTGGCTGACAGTTTCTCCAAGG    1060
Patas           TGGGTTTGGGGTAGGGTCGGTTCTATCTGTTCTACTCTGTGGCTGACAGTTTCTCCAAGG    1079
AGM             TGGGTTTGGGGTAGGGTCGGTTCTATCTGTTCTACTCTGTGGCTGACAGTTTCTCCAAGG    1079
                * * ******** **************** ** ***************************
                      ZEB
Human           GGCTGCAGGTGTCAGCTGTCTGAGCCCGGGCTGAGCTCTGAAACGTGCCTACTCAAACTT    1139
Rhesus          GGCTGCAGGTGTCAGCTGTCTGAGGCCAGCCTGAGCTCTGAAATGTGCCTACTCAAACTT    1120
Patas           GGCTGCAGGTGTCAGCTGTCTGAGCCCAGCCCGAGCTCTGAAATGTGCCTACTCAAACTT    1139
AGM             GGCTGCAGGTGTCAGCTGTCTGAGCCCAGCCTGAGCTCTGAAATGTGCCTACTCAAACTT    1139
                ************************ ** * * *********** ****************

Human           CCCGTGGGGTAGGGGAGGCCCAGAACCACCCTCTGAGAGTGGCAAAAAGTGGTCCTGGAG    1199
Rhesus          CCA-TGGGGTGAGGGAGGCCCAGAACCACCCTCTGAGAGTGGCAACAAGTGGTCCTGCAG    1179
Patas           CCCGTGGGGTGAGGGAGGCCCAGAACCACCCTCTGAGAGTGGCAACAAGTGGTCCTGCAG    1199
AGM             CCCGTGGGGTGAGGGAGGCCCAGAACCACCCTCTGAGAGTGGCAACAAGTGGTCCTGCAG    1199
                **  ******  ********************************* *********** **

Human           CCAGGGGAAATGTGGATGGGGTAGA-1224
Rhesus          CCAGGGGAAATGTGGATGGGGTAGAC1205
Patas           CCAGGGGAAATGTGGATGGGGTAGAC1225
AGM             CCAGGGGAAATGTGGATGGGGTAGAC1225
                ************************
```

```
CLUSTAL O(1.2.4) multiple sequence alignment: TSS

                                                                ELF/ETS
Patas           ----GGGGCCGGCCCAGGCCTAGGGTGTGGAGGAACCTCGCCATCGGGCTTCCTGTCTCT    56
AGM             ----GGGGCCGGCCCAGGCCTAGGGTATGGAGGAGCCTCGCCATCGGGCTTCCTGTCTCT    56
Rhesus          ----GGGGCCGGCCCAGGCCTAGGGTGTGGAGGAGCCTCGCCATCGGGCTTCCTGTCTCT    56
Human           GTTTGGGGCCGGCCCAGGCCTAGGGTGTGGAGGAGCCTTGCCATCGGGCTTCCTGTCTCT    60
                    ********************** ******* *** *********************
                                              TCF-1
Patas           CTTCATTTAAGCACGACTCTGCAGAAGGAACAAAGCACCCTCCCCACTGGGCTCCTGGTT    116
AGM             CTTCATTTAAGCACGACTCTGCAGAAGGAACAAAGCACCCTCCCCACTGGGCTCCTGGTT    116
Rhesus          CTTCATTTAAGCACGACTCTGCAGAAGGAACAAAGCACCCTCCCCACTGGGCTCCTGGTT    116
Human           CTTCATTTAAGCACGACTCTGCAGAAGGAACAAAGCACCCTCCCCACTGGGCTCCTGGTT    120
                ************************************************************

Patas           GCAAAGCTCCAAGTCCTCACACAGATACACTTGT  150
AGM             GCAAAGCTCCAAGTCCTCACACAGATACACTTGT  150
Rhesus          GCAAAGCTCCAAGTCCTCATACAGATACACTTGT  150
Human           GCAGAGCTCCAAGTCCTCACACAGATACGC----  150
                *** *************** ******** *
```

CLUSTAL O(1.2.4) multiple sequence alignment: S4

```
Human       CGGTTCCCCCGATGTGGGTGTCTGAGGCGAAGAAGAGGATGGCGGAGGTTGCAGCCACCA    60
Rhesus      CGGTTCCCCCGATGTGGGTGTCTGAGGCGAAGAAGAGGATGGCGGAGGTTGCAGCCACCA    60
Patas       CGGTTCCCCCGATGTGGGTGTCTGAGGCGAAGAAGAGGATGGCGGAGGTTGCAGCCACCA    60
AGM         CGGTTCCCCCGATGTGGGTGTCTGAGGCGAAGAAGAGGATGGCGGAGGTTGCAGCCACCA    60
            ************************************************************

             RUNX3
Human       ACCACAAGAGTTCCTTAGAGGGGTCACAGTCTCTAGGAAGTTTATAGGAAGCTAGTCAGC    120
Rhesus      ACCACAAGAGTTCCTTAGAGGGGTCACAGTCTCTAGGAAGTTTATAGGAAGCCAGTCAGC    120
Patas       ACCACAAGAGTTCCTTAGAGGGGTCACAGTCTCTAGGAAGTTTCTAGGAAGCCAGTCAGC    120
AGM         ACCACAAGAGTTCCTTAGAGGGGTCACAGTCTCTAGGAAGTTTCTAGGAAGCCAGTCAGC    120
            ******************************************* ******** *******

Human       AGTAGAGAGGGTGAACGCGGTGGGGCACATCCCGCGGCTGGGCTTGAGTGGGCTGCTTGG    180
Rhesus      AGTAGAGAGGGTGAACGCGGTGGGGCACATCCCGCGGCTGGGCTTGAGTGGGCTGCTTGG    180
Patas       AGTAGAGAGGGTGAACGCGGTGGGGCACATCCCGCGGCTGGGCTCGAGTGGGCTGCTTGG    180
AGM         AGTAGAGAGTGTGAACGCGGTGGGGCACATCCCGCGGCTGGGCTCGAGTGGGCTGCTTGG    180
            ********* ********************************** ***************

                              LEF1
Human       GGGTTATGGGGAGAAGATAAAAGTGCCTGTGGGACCACAGACTCTCGCTGTGGTGGAGCT    240
Rhesus      GGGTTATGGAGAGAAGATAAAAGTGCCTGTGGGACCACAGACTCTTGCTGTGGTGGAGCT    240
Patas       GGGTTATGGAGAGAAGATAAAAGTGCCTGTGGGACCACAGACTCTCGCTGTGGTGGAGCT    240
AGM         GGGTTATGGAGAGAAGATAAAAGTGCCTGTGGGACCACAGACTCTCGCTGTGGTGGAGCT    240
            ********* *********************************** **************

Human       GGGCCCTCTTACCCTCCCAAGCCTCGCCCCTCATCCCATCCCTGGGGGCCAGGGGTGAGG    300
Rhesus      GGGCCCTCTTACCCTCCCAAGCCTCGCTCCTCATCCCATCCCTGGGGGCCAGGGGTGAGG    300
Patas       GGGCCCTCTTACCCTCCCAAGCCTCGCTCCTCGTCCCATCCCTGGGGGCCAGAGGTGAGG    300
AGM         GGGCCCTCTTACCCTCCCAAGCCTCACTCCTCGTCCCATCCCTGGGGGCCAGGGGTGAGG    300
            ************************* * **** ******************* *******

                     RUNX3
Human       GCGGCAGGAACCTCAAGGCTCTGAGAAAGTGCGTGGTGTGTGTTGCCATTTTGGTCTCTT    360
Rhesus      GCGGCAGGAACCTCAAGGCTCTGAGAGAGTGCGTGGTATGTGTTGCCATTTTGGTCTCAT    360
Patas       GCGGCAGGAACCTCAAGGCTCTGAGAGAGTGCGTGGTGTGTGTTGCCATTTTGGTCTCAT    360
AGM         GCGGCAGGAACCTCAAGGCTCTGAGAGAGTGCGTGGTGTGTGTTGCCATTTTGGTCTCAT    360
            ************************** ********** ******************** *

Human       CTCTTTCTCAGTCTCTCTTTGCCTCACTTTGGATCTATGCTCTGTGCATCTGTCTT-       416
Rhesus      CTCTTTCTCAGTCTCTCTTTGCCTCACTTTGGATCTATGCTCTGTGCATCTGTCTTG       417
Patas       CTCTTTCTCAGTCTCTCTTTGCCTCACTTTGGATCTGTGCTCTGTGCATCTGTCTTG       417
AGM         CTCTTTCTCAGTCTCTCTTTGCCTCACTTTGGATCTGTGCTCTGTGCATCTGTCTTG       417
            ************************************ *******************
```

```
CLUSTAL O(1.2.4) multiple sequence alignment: E4M

                                         ELK/ETS
Human           AGGCCAGGAGGACTGTGTACAGACCGGAAGGAGCTAGAGCTTAGTGGCAGCCTGAGAGGG     60
Rhesus          -GGCCAGGAGGCCCGTGTACAGACCGGAAGCAGCTAGAGCTTAGTGGCAGCTGGAGAGGG     59
Patas           -GGCCAGGAGGCCTGTGTACAGACCAGAAGCAGCTAGAGCTTAGTGGCAGCTGGAGAGGG     59
AGM             -GGCCAGGAGGCCTGTGTACAGACCGGAAGCAGCTAGAGCTTAGTGGCAGCTGGAGAGGG     59
                 ********** * *********** **** ********************  *******

Human           GAAGCTGAAAAAGGAGAAGAGGCAAGGGGCATTCCAGGGGAGCCCGGGAGAGCCAGCACG     120
Rhesus          GAAGATTATAAAGGAGGAGAGGCAAGGGGCATTCCAGGGGAGCCCGGGAGAGCCAGCATG     119
Patas           GAAGATTATAAAGGAGGAGAGGCAAGGGGCATTCCAGGGGAGCCTGGGAGAGCCAGCACG     119
AGM             GAAGATTATAAAGGAGGAGAGGCAAGGGGCATTCCAGGGGAGCCTGGGAGAGCCAGCACG     119
                **** * * ******* *************************** ************* *

Human           GCCGCCTGGTATATGAGGCAAAGAGGAAGACAGACACAGACACAGGGAGCTGCAGGCTGG     180
Rhesus          ACCTCCTGGCATATGAGGCACAGAGGAAGACAGACACAGACACAGGGAGCTGCAGGCTGG     179
Patas           ACCTCCTGGCATATGAGGCACAGAGGAAGACAGACACAGACACAGGGAGCTGCAGGCTGG     179
AGM             ACCTCCTGGCATATGAGGCACAGAGGAAGACAGACACAGACACAGGGAGCTGCAGGCTGG     179
                 ** ***** ********** ***************************************

Human           GGGCATAAGCTGGGGGCTGGGAAGCATAGATACAGAAATGCACAGATGTGAGCTGAGAAG     240
Rhesus          GGGCATAAGCTGGGGGCTGGGAAGCATAGATACAGAAATGCACAGATGTGAGCTGAGAAG     239
Patas           GGGCATAAGCTGGGGGCTGGGAAGCACAGATACAGAAATGCACAGATGTGAGCTGCGAAG     239
AGM             GGGCATAAGCTGGGGGCTGGGAAGCACAGATACAGAAATGCACAGATGTGAGCTGAGAAG     239
                ************************** **************************** ****

Human           CAAGGAGGGAGAGAGAGAG----ACAGAAAGAGAGAGAGAGACGTGCCAGGGCTTGAGGG     296
Rhesus          CAAGCAGAGAGAGAGAGAGAGAGAGAGAGAGAAAGAGAGAGACGTGCTAGGGCTTGAGGG     299
Patas           CAAGCAGAGAGAGAGAGAGA------GAGAGAGAGAGAGAGACGTGCTAGGGCTTGAGGG     293
AGM             CAAGCAGAGAGAGAGAG------------AGAGAGAGAGAGACGTGCTAGGGCTTGAGGG     287
                **** ** *********            *** ************** ************

Human           ACCAGAGAGCCCTCCCAGCCTCTCTCGGAGTGCTGGTATACAGGATGCTACCGTACTAGG     356
Rhesus          ACCAGAGAGCCCTCCCAACCTCTCTCGGAGTGCTGGTCTACAGGATGCTGCTGTACTAGC     359
Patas           ACCAGAGAGCCCTCCCAACCTCTCTCGGAGTGCTGGTCTACAGGATGCTACCGTACTAGG     353
AGM             ACCAGAGAGCCCTCCCAACCTTTCTCGGAGTGCTGGTCTACAGGATGCTACCGTACTAGG     347
                ***************** *** *************** *********** * *******
                                                      TCF/LEF
Human           GTAAGACACCTCTGGGGACGCTGAGTATGGGAATCAAAGGCCAGATCTCTGGGGTGGCAG     416
Rhesus          GTAAGGCACCTCTGGGGACGCTGAGTATGGGAATCAAAGGCCGGATCTCTGTGGTGGCAG     419
Patas           GTAAGGCACCTCTGGGGACGCTGAGTATGGGAATCAAAGGCCAGATCTCTGCGGTGGCAG     413
AGM             GTAAGGCACCTCTGGGGACGCTGAGTATGGGAATCAAAGGCCAGATCTCTGCAGTGGCAG     407
                ***** ************************************ ********  *******
                                                           RUNX1
Human           CGGAAGCCCAAAGCACCAAAGCAAGCATGCTGGAAACCCACAGCCTCCTCCACTTAGCAG     476
Rhesus          CGGAAGCCCAAAGCACCAAAGCAAGCATGCTGGAAACCCACAGCCTCCTCCACTTAGCAG     479
Patas           CGGAAGCCCAAAGCACCAAAGCAAGCATGCTGGAAACCCACAGCCTCCTCCACTTAGCAG     473
AGM             CGGAAGCCCAAAGCACCAAAGCAAGCATGCTAGAAACCCACAGCCTCCTCCACTTAGCAG     467
                ******************************* ****************************

Human           AGCCTTGGGGTGAGATGAGGCAGAACAGGGAGCTGGAGGCAGGGAGGTGGCTGTCTGCAC     536
Rhesus          AGCCTTGGGGTGAGATGAGGCGGAACAGGGAGCTGGAGGCAGGGAGGTGGCTGTCTGCAC     539
Patas           AGCCTTGGGGTGAGATGAGGCGGAACAGGGAGCTGGAGGCAGGGAGGTGGCTGTCTGCAC     533
AGM             AGCCTTGGGGTGAGATGAAGCGGAACAGGGAGCTGGAGGCAGGGAGGTGGCTGTCTGCAC     527
                ****************** ** **************************************

Human           ATACCTCAGGACCATGGAGCTGGGGGAGTCAAAACAGCCACCATATGGGGAAGGGTCAAG     596
Rhesus          AGACCTCAGGACCATGGAGCAGGGGGAGTCAAAACAGCCACCATGTGGGGAAGGGTCAAG     599
Patas           AGACCTCAGGACCATGGAGCAGGGGGAGTCAAAACAGCCACCATGTGGGGAAGGGTCAAG     593
AGM             AGACCTCAGGACCATGGAGCAGGGGGAGTCAAAACAGCCACCATGTGGGGAAGGGTCAAG     587
                * ****************** *********************** ***************

Human           AATGCCTCTAGTCTTCCCCAGGCATCTTATCAGGGTAAGCTGAATTTGGACCCCAGAGAA     656
Rhesus          AATGCCTCTAGTCTTCCCCAGGCATCTTATCAGGGTAAGCTGGATTTGGACCCCAGAGAA     659
Patas           AATGCCTCTAGTCTTCCCCAGGCATCTTATCAGGGTAAGCTGGATTTGGACCCCAGAGAA     653
AGM             AATGCCTCTAGTCTTCCCCAGGCATCTTATCAGGGTAAGCTGGATTTGGACCCCAGAGAA     647
                ****************************************** *****************

Human           GGGATATCGTTTATGGAGACTTCCCCTCTTTCATCCCCTGCTCACCAAGGACC  709
Rhesus          GGGATATCCTATATGGAGACTTCCCCTCTTTCACTCCCTGTTCACCAAG----  708
Patas           GGGATATCCTATATGGAGACTTCCCCTCTTTCACTCCCTGCTCACCAAG----  702
AGM             GGGATATCCTATATGGAGACTTCCCCTCTTTCACTCCCTGCTCACCAAG----  696
                ******** * **********************  ***** ********
```

Supplementary figure 1. DNA sequence alignments determined by ClustalX of CD4 regulatory regions in humans, rhesus macaque, Patas, and AGM. Bases highlighted in yellow represent sequences conservation in natural host species that have diverged from progressive hosts of HIV-1/SIV. Regions denoted in red represent transcription factor binding motifs predicted from the JASPAR database.
